# White’s Operation for Enlarged Prostate

**Published:** 1894-03

**Authors:** Francis L. Haynes

**Affiliations:** Los Angeles, Cal.; 929 South Main Street


### Clinical Memorandum.

### WHITE'S OPERATION FOR ENLARGED PROSTATE.

By FRANCIS L. HAYNES, M. D., Los Angeles, Cal.

IN THE last number of the JOURNAL was published a very interesting discussion of the treatment of prostatic hypertrophy. Allusion was made to the operation for its cure, devised by Prof. J. William White, of Philadelphia, (castration,) which has now been performed at least four times in this country.

The first operation (by myself) was made eight weeks ago. The patient seems to be practically cured.

Two weeks since, I made White's operation on a very aggravated case. The patient stated that he had passed no urine, except through the catheter, for two years. Since then he has daily passed several ounces, and yesterday more than a pint.

I am informed that two operations have been made in addition to these, but as it is probable that the originator of the operation will publish them, I will not further allude to them.

I do not doubt that when sufficient experience has demonstrated the place of this operation, it will prove as beneficent as the analogous operation has proven in appropriate cases of uterine myoma.

It is to be hoped that hereafter patients with enlarged prostate will not be allowed to reach a desperate condition, but will by an early operation be granted a fair chance for many years of comfortable existence.

929 SOUTH MAIN STREET.

---

TANNIN AND BORIC ACID IN DYSENTERY.—Liebersohn (*Vratch,*) reports two cases of severe acute dysentery which were treated by hot enemata of tannin and boric acid. The results seems to show that the injection speedily arrested the intestinal hemorrhage and quickly restored the natural character of the passages. Pain and tenesmus were immediately relieved and the course of the disease materially shortened. The enemata were given every three hours, each consisting of one fluid pound of a four per cent. solution of boric acid, ten grains of tannin, and three and three-quarter drops of tincture of opium, the whole to be dissolved in a tumbler and a half of hot boiled water. The injecting fluid was retained in the bowel for one or two minutes.—*University Medical Magazine.*

